# *Strongyloides ratti* and *S. venezuelensis* – rodent models of *Strongyloides* infection

**DOI:** 10.1017/S0031182016000020

**Published:** 2016-03-03

**Authors:** MARK VINEY, TAISEI KIKUCHI

**Affiliations:** 1 School of Biological Sciences, University of Bristol, Bristol BS8 1TQ, UK; 2 Division of Parasitology, Faculty of Medicine, University of Miyazaki, Miyazaki, Japan

**Keywords:** *Strongyloides*, rats, rodent

## Abstract

*Strongyloides* spp. are common parasites of vertebrates and two species, *S. ratti* and *S. venezuelensis*, parasitize rats; there are no known species that naturally infect mice. *Strongyloides ratti* and *S. venezuelensis* overlap in their geographical range and in these regions co-infections appear to be common. These species have been widely used as tractable laboratory systems in rats as well as mice. The core biology of these two species is similar, but there are clear differences in aspects of their within-host biology as well as in their free-living generation. Phylogenetic evidence suggests that *S. ratti* and *S. venezuelensis* are the result of two independent evolutionary transitions to parasitism of rats, which therefore presents an ideal opportunity to begin to investigate the basis of host specificity in *Strongyloides* spp.

## INTRODUCTION

The genus *Strongyloides* contains some 60 species that infect a wide range of mammals, birds, reptiles and amphibians. Two species, *S. ratti* and *S. venezuelensis*, parasitize brown rats (*Rattus norvegicus*) and, largely for this reason, have been domesticated for laboratory study in rodent hosts. No species of *Strongyloides* has been described from any species of mouse, despite the extensive parasitological interest directed at such hosts. Both *S. ratti* and *S. venezuelensis* can experimentally infect laboratory mice (*Mus musculus domesticus*), though not as easily compared with their infection of rats (Dawkins *et al.* 1983; Sato and Toma, 1990; Amarante and Oliveira-Sequeira, 2002).

## NATURAL HISTORY

*Strongyloides ratti* was first described from rats caught on a Baltimore (USA) rubbish dump (Sandground, 1925) though earlier work also reported *Strongyloides* sp. from *R. norvegicus* in Italy (reported in Sandground, 1925). *Strongyloides ratti* appears to be widespread (if not ubiquitous) around the world, occurring wherever rats are present. All *S. ratti* infections to date have been recorded from *R. norvegicus* rather than other *Rattus* spp., but this finding, at least in part, likely reflects sampling bias.

*Strongyloides venezuelensis* was first described from rats in Venezuela (Brumpt, 1934). This description was brief and incomplete, so the species was then redescribed from *R. norvegicus* in the USA, this time including a description of the free-living generation (Little, 1966). *Strongyloides venezuelensis* is widely distributed in warm regions of the world, and in addition to its original descriptions it has been reported from Israel, Brazil and Japan (Wertheim and Lengy, 1964; Araujo, 1967; Hasegawa *et al.* 1988). All of the locations from which it has been reported are between the 35° north and south parallels, suggesting that it requires a warm climate for development in the external environment (Hasegawa *et al.* 1988). All the reports of *S. venezuelensis* are from brown rats (*R. norvegicus*). *Strongyloides ratti* and *S. venezuelensis* therefore infect the same host species though *S. venezuelensis* has a more limited climatic range compared with *S. ratti*.

There is little information on the prevalence or intensity of wild infections since most reports of infection are anecdotal. For *S. ratti* the original species description reported a prevalence of about 60% (Sandground, 1925). One study of wild rats in the UK found that the prevalence was 62% (sample size 111) (Fisher and Viney, 1998). These latter infections were diagnosed by faecal culture, which is a more sensitive diagnostic method compared with identification of eggs or larvae in faeces. These data also showed that there was a range of infection intensities, probably reflecting the well-known overdispersed distribution of macroparasites. For *S. venezuelensis* the prevalence can be similarly high, reported as 71 and 100% in 14 and 8 rats in New Orleans and Japan, respectively (Little, 1966; Kikuchi, unpublished observations), though at 25% among 431 rats in Israel (Wertheim and Lengy, 1964).

*Strongyloides ratti* and *S. venezuelensis* naturally co-infect hosts (Little, 1966; Wertheim, 1970*a*; Hasegawa *et al.* 1988); for example, in wild rats in Israel co-infection was common with an overall *Strongyloides* prevalence of 36%, but among the infected rats 45% of infections were *S. ratti* and *S. venezuelensis* co-infections (Wertheim and Lengy, 1964). In experimental co-infections, there are synergistic effects between the two species such that the infection success of each species is greater in co-infections compared with in single-species infections (Wertheim, 1970*b*). The intestinal position (both longitudinal and radial) of each species did not differ between co-infections and single-species infections (Wertheim, 1970*b*).

## COMPARATIVE MORPHOLOGY

The morphology of *S. ratti* and *S. venezuelensis* are similar, but there are a few key characters that differentiate them. The principal morphological difference between them is in the arrangement of the ovaries and intestine in the parasitic females: in *S. ratti* the ovaries lie parallel to the intestine, whereas in *S. venezuelensis* they are intertwined (Little, 1966). In addition to this feature, the shape of the parasitic female stoma and the stage passed in the faeces of the host also differentiates the species. The stoma of the parasitic female of *S. venezuelensis* is ‘ornate’, whereas for *S. ratti* it is ‘badge-shaped’; for the stage passed in faeces, for *S. ratti* this is a mixture of eggs and larvae whereas for *S. venezuelensis* only early stage eggs are passed (Little, 1966). (To observe this latter character, fresh faeces must be examined because eggs rapidly develop and hatch once passed from a host.) The distance from the cephalic apex to the nerve ring and to the excretory pore is also reported to differentiate these species (Hasegawa *et al*. 1988). In summary, there are a number of characters of the parasitic female that can easily distinguish these species. Importantly though, there is no reliable way to distinguish the free-living stages of these species (Little, 1966). The conditions in which the free-living stages are grown and the within-host conditions can affect the morphology of the free-living and parasitic generation of *Strongyloides* spp., respectively (Speare, 1989), and this has been shown specifically for the infective third stage larvae (iL3s) of *S. venezuelensis* (Islam *et al*. 1999).

In addition to these morphological differences, the species have different karyotypes (*S. ratti* 2*n* = 6, *S. venezuelensis* 2*n* = 4), and a molecular phylogeny of *Strongyloides* show that they belong to two distinct groups (Hino *et al*. 2014; Hunt *et al.* 2016). In one group are *S. ratti* and the parasite of humans *S. stercoralis*, and the other *S. venezuelensis, S. papillosus* and *S. fuelleborni* (a parasite of humans and other primates). Together this suggests that *Strongyloides* has independently evolved parasitism of rats twice (Hino *et al*. 2014; Hunt *et al.* 2016).

## HOST INFECTION AND WITHIN-HOST MIGRATION

*Strongyloides ratti* and *S. venezuelensis*, as with all *Strongyloides* spp., infect hosts by their iL3s penetrating host skin. Experimental *S. ratti* infections where the iL3s were applied to the skin and left to penetrate naturally showed that most larvae (70%) penetrate the skin within 5 min (Tindall and Wilson, 1988). For *S. venezuelensis* skin penetration is completed 10–20 min after exposure (Wertheim, 1970*a*).

The within-host migration route of *S. ratti* has been studied in detail, which has rigorously demonstrated that larval migration *via* the naso-frontal part of the head is at least one route to the host gut (Tindall and Wilson, 1988). For *S. venezuelensis* after skin penetration larvae are found throughout the body, in the skin, muscles and lung, which has suggested that these sites are all part of the route of migration to the gut (Takamure, 1995). Almost no larvae were found in the head region, suggesting that *S. venezuelensis* has a different within-host migration route compared with *S. ratti*.

Both *S. ratti* and *S. venezuelensis* can be transmitted transmammarily (Nolan and Katz, 1981; Wilson and Simpson, 1981; Kawanabe *et al*. 1988). The window of opportunity for transmammary transmission of *S. venezuelensis* appears to be small, occurring when a mother becomes infected shortly after giving birth (Nolan and Katz, 1981). For *S. ratti*, considerable evidence has been presented supporting the idea that migrating larvae are actively diverted to the mammary glands when a mother is suckling (Wilson and Simpson, 1981).

## LABORATORY MAINTENANCE

Both *S. venezuelensis* and *S. ratti* are easy to maintain in the laboratory. Rats can be infected by a subcutaneous injection of iL3s, and the infections are then patent from 4 to 5 days post infection (dpi) (Takamure, 1995). If desired, a more natural style infection can be used where iL3s are applied to the animal's skin. This is most usually done by shaving a small patch of skin of an anaesthetized rat before applying the larvae to the skin (Tindall and Wilson, 1988).

*Strongyloides ratti* and *S. venezuelensis* have different efficiencies at infecting the host and/or different effects on the host. For *S. ratti*, 500 iL3s are typically used to initiate an infection in a rat and this has no overt clinical effect; a dose of *c.* 5000 iL3s would be the typical maximum dose that could be tolerated by a rat. This, though, does vary depending on the source of *S. ratti*; for example, a recent wild isolate of *S. ratti* was severely pathological to rats at a dose of 500 iL3s (Hunt and Viney, unpublished observations). In contrast, with *S. venezuelensis* doses of up to 30 000 iL3s are used and this does not appear to be overtly harmful to the rats (Taira *et al*. 1995). Although there is limited information, it would appear that *S. venezuelensis* and *S. ratti* infect with similar efficiencies. For single iL3 infections of *S. ratti* approximately two-thirds of infections become patent (Viney, unpublished observations), whereas for *S. venezuelensis* about half do (Kikuchi, unpublished observations). Extrapolating from this further suggests that the very large doses of *S. venezuelensis* iL3s given to rats results in similarly large intensities of infection, therefore suggesting that *S. venezuelensis* is less pathogenic to rats than *S. ratti.*

Once animals are infected, then faeces can be collected from them and this used as the source for growing the free-living generation. For *S. ratti* and *S. venezuelensis* this can be simply done by maintaining the faeces damp and at an ambient, or slightly elevated, temperature. The free-living stages then grow in the faeces and infective larvae are seen, typically migrating away from the faecal mass. This development is swift. For *S. ratti*, in faecal cultures maintained at 19 °C, free-living males and females are present after 2 days of incubation, and their iL3 progeny are present the next day. For *S. venezuelensis*, free-living males and females are rarely observed in the laboratory cultures maintained at 25 °C. This means that only directly developing iL3s are produced in cultures, with these larvae being present after 2 days. More detailed methods are available in (Viney and Lok, 2015).

Multiple strains of laboratory rats appear to be similarly susceptible to *S. venezuelensis*, though the age and sex of Wistar rats does affect the dynamics of infection, such that female rats become more resistant to infection as they age, whereas male rats become more susceptible to infection with age (Takamure, 1995; Baek *et al*. 1998; Rivero *et al*. 2002; Marra *et al*. 2011). Laboratory mice have been also been widely used to study *S. venezuelensis* infections, and mouse strains differ substantially in their susceptibility to infection. For example, C57BL/6 mice are highly susceptible to *S. venezuelensis* infection, while BALB/c, C3H/He and NIH are more resistant (Sato and Toma, 1990; Amarante and Oliveira-Sequeira, 2002).

Other host species that are susceptible to *S. venezuelensis* are Mongolian gerbils *Meriones unguiculatus* (Horii *et al.* 1993; Tsuji *et al.* 1993; Baek *et al.* 2002), Syrian golden hamsters *Mesocricetus auratus* (Shi *et al.* 1994), Indian soft-furred rats, *Millardia meltada* (Tiuria *et al.* 1995) and cotton rats, *Sigmodon hispidus* (Attamimi *et al.* 2002).

Because parasitic females reproduce by mitotic parthenogenesis (shown formally for *S. ratti*, but presumed to be true for *S. venezuelensis* too; Viney, 1994), isofemale lines (i.e. lines derived from a single parasitic female) can be made. This is done by injecting a single iL3s into a rat and searching for progeny in faeces (Graham, 1938*a*). All three life-cycle morphs (free-living males, free-living females and directly developing iL3s) can develop from a single parasitic female, and so an isofemale line can be established from a single, diploid *Strongyloides* genome. If an isofemale line is maintained by passage of directly developing iL3s (and thus without the sexual reproduction of the free-living adults), then this will genetically maintain the original diploid genome of the foundress parasitic female. If the isofemale line is maintained via free-living adults, then their sexual reproduction will lead to loci becoming homozygous.

Laboratory maintenance of any parasite can significantly affect important life-history traits (Viney, 2013). In *S. ratti*, this has been studied explicitly by maintaining infections for up to 50 generations derived from either iL3s that developed early in infection or from those that developed late in infection (Paterson and Barber, 2007). This selection resulted in differences in the fecundity of the lines, particularly how their fecundity changed because of density-dependent effects over the course of an infection (Paterson and Barber, 2007).

## THE BIOLOGY OF THE PARASITIC FEMALES

The parasitic females of both species live in the rat small intestine. In laboratory *S. ratti* infections, parasitic females are concentrated in the *c.* 30 cm of the intestine distal to the rat's stomach (Moqbel and Denham, 1977; Korenaga *et al.* 1983; Kimura *et al*. 1999; Wilkes *et al*. 2004). *Strongyloides venezuelensis* has a somewhat more anterior distribution, being concentrated in the proximal most 15 cm of the gut, with over half of worms in the first 5 cm distal to the pylorus (Wertheim, 1970*b*; Hasegawa *et al*. 1988). However, these positions are dynamic. For *S. ratti*, as the infection progresses and an anti-*S. ratti* immune response develops, the parasitic females become more spread out (though maintaining the same anterior extent) along the intestine. Later still in an infection some parasitic females move to the caecum and colon where they continue to reproduce (Kimura *et al.* 1999). Presumably this change in position in the gut is a way for the worms to continue to maximize their fitness as the host immune response makes their current niche less desirable; for example, this distal move may be a way to escape from a localized mucosal immune response. This extreme distal migration of parasitic females was seen in very high-intensity laboratory infections, so whether such phenomena occur in natural low-intensity infections is not known (Kimura *et al.* 1999). Of note, *S. venezuelensis* will establish in the ileum if it is directly implanted there (Maruyama *et al.* 2002).

*Strongyloides* spp. is a tissue-dwelling, rather than luminal-dwelling, parasite. *Strongyloides ratti* is found in the intestinal mucosa epithelium, though there are some reports of it in the crypts of Lieberkühn (Dawkins *et al*. 1983; Hasegawa *et al.* 1988). Studies of *S. ratti* in mice found that a single parasitic female was mainly in the epithelium across up to four villi (with occasionally some portions of the body protruding into the lumen), and sometimes close to the villus crypts (Dawkins *et al*. 1983). *Strongyloides venezuelensis* is also found in the luminal surface of the intestinal mucosa (Araujo, 1967; Wertheim, 1970*b*). For both species, the parasitic females appear to continuously migrate through intestinal tissue, such that histological descriptions of their precise position within and between villi should perhaps be treated with some caution. (Also, given that the host immune status also affects the longitudinal positioning of *S. ratti* in the intestine, parasitic females’ radial position may similarly be affected by these factors.) However, the consequence of this intra-mucosal migration is that the worms build a mucosal tunnel among the mucosal cells, along which the worms migrate, laying clumps of eggs as they go (Dawkins *et al*. 1983; Maruyama *et al.* 2000). A vacuole often appears to form around the worms, being delimited by a continuous host cell membrane, suggesting that the worm tunnels are fluid-filled (Dawkins *et al*. 1983).

The biology of these tunnels is rather unexplored (but fascinating). It is likely that the worms are feeding on host tissue as they migrate, and so the tunnel is the result of them excavating their food. Indeed, the worm's head appears to be in close contact with epithelial cells (Dawkins *et al*. 1983). Because the host mucosa continually turns over (and this is perhaps speeded by the *Strongyloides*’ burrows), when the mucosa sloughs off and is passed out of the host, *Strongyloides* eggs and larvae pass out of the host too.

*In vitro* studies with *S. venezuelensis* have shown that parasitic females release copious amounts of a sticky product from their mouths (produced from oesophageal glands) with which they adhere to laboratory plastic ware (Maruyama and Nawa, 1997; Maruyama *et al.* 2003); it seems likely that the biology of *S. ratti* will be similar in this respect. These products were glycosylated, were distinct from rat mucins, and were recognized by *S. venezuelensis-*infected rat sera (Maruyama and Nawa, 1997). *In vivo* these secretions presumably fill the tunnel ahead of the migrating worms and line the tunnel walls (as shown by antibodies against these products staining the tunnel lining; Maruyama *et al.* 2003). Genomic and proteomic analyses of *S. ratti* suggest that it secretes large quantities of proteases and other gene products, which presumably contribute to these secretions (Hunt *et al.* 2016). Direct proteomic analysis of the *S. venezuelensis* products shows that trypsin inhibitor like (TIL) domain containing proteins, as well as some proteases, are likely to be the main components of these products (Kikuchi, unpublished observations). These TIL domain containing proteins appeared to be highly O-glycosylated, which probably contributes to the stickiness of these secretions. Again, it seems likely that these secretions (combined with the worms’ forward movement) is what is excavating the mucosal tunnels (Maruyama *et al.* 2003; Hunt *et al.* 2016). But further, the concentration of these worm-derived molecules in these tunnels may be creating a physiological niche for the parasite, one that is distinct from the surrounding host mucosa.

## THE EFFECTS OF THE HOST IMMUNE RESPONSE

The immunobiology of *Strongyloides* is being considered elsewhere in this volume, so here we address the biological effect of the host immune response on *S. ratti* and *S. venezuelensis.*

Laboratory infections of rats with *S. ratti* and *S. venezuelensis* provoke an immune response so that infected rats expel most of the parasites by about 10–14 dpi.

After worms are expelled, rats are strongly immune to reinfection, seen as diminished worm burdens, decreased egg output and rapid expulsion of a challenge infection (Sato and Toma, 1990; Korenaga *et al.* 1991; Wilkes *et al*. 2007). In common with most helminths (Finkelman *et al.* 1997), the rat anti-*S. ratti* immune response is a T-helper type 2 (Th2) response (Wilkes *et al*. 2007). But rat anti-*S. ratti* immune responses are parasite dose-dependent (Uchikawa *et al*. 1991). At low doses this is a T-helper type 1 (Th1) response which is ineffective against *S. ratti*, only becoming an effective Th2 response at higher parasite doses (Bleay *et al*. 2007). The magnitude of the Th2 response is also dose dependent (Bleay *et al*. 2007). Put simply, the dose of *S. ratti* administered to a rat affects the qualitative and quantitative nature of the host anti-*S. ratti* immune response.

*Strongyloides ratti* is also subject to immune-dependent density-dependent effects that affect the establishment, subsequent survival and fecundity of the parasitic females (Paterson and Viney, 2002). These phenomena have not been investigated for *S. venezuelensis*.

Parasitic females change their intestinal position (above), shrink and become less fecund in the presence of an anti-*S. ratti* immune response (Moqbel and Denham, 1977; Wilkes *et al*. 2004). These effects can be reversed by transplantation to naïve hosts or by host immunosuppression (Moqbel *et al*. 1980; Viney *et al.* 2006). Also, when *S. ratti* parasitic females move to an extreme distal position in the host gut, they appear to recover some of the immune-dependent reduction in their size and fecundity (Kimura *et al.* 1999). This phenomenon whereby parasitic nematodes shrink and lower their fecundity because of the host immune response is known from other nematode infections too (Stear *et al*. 1997). The niche of *S. ratti*, and presumably *S. venezuelensis* too, parasitic females are therefore dynamic, largely being driven by the host immune response.

There are also other effects of the host immune response that have been studied in *S. venezuelensis* (Moqbel *et al.* 1980). As parasitic females are exposed to the host immune response their intestinal tissue is damaged, and their gut lumen is often empty. The mouth and peri-oral region of the parasitic females become clogged with immunoglobulin-containing material (Moqbel *et al.* 1980). Together, these observations suggest that the host immune response progressively prevents the worms from feeding (and likely their ability to build tunnels in host tissue), which presumably underlies their reduced size and fecundity (Moqbel and McLaren, 1980).

Putting these observations together – the *S. ratti* and *S. venezuelensis* parasitic females burrow through the host mucosa, with this aided by the parasites’ oral secretion of enzymes and other molecules, and that these molecules are immunogenic, and the host immunoglobulins bind these secretions blocking the mouth of the parasitic females. This interferes with the feeding of the parasite (both because the parasite-derived molecules are functionally neutralized by host immunoglobulin and because these immunoglobulin-containing complexes physically block the mouth of the parasitic females) with the result that the worms shrink, become less fecund, and also change their position in the host gut.

The immunological responses of wild rats naturally infected with *S. ratti* have not been investigated. However, it is likely that they are substantially different compared with those of laboratory rats, for the following reasons. In laboratory rats, *S. ratti* and *S. venezuelensis* infection generate an immune response that eliminates worms from rats and makes them strongly resistant to reinfection (Moqbel and Denham, 1977; Moqbel *et al.* 1980; Harvey *et al*. 2000). If such processes occurred in wild *Strongyloides*-infected rats, then *S. ratti* and *S. venezuelensis* would likely be rare infections, perhaps only existing as epidemics when sufficient *Strongyloides*-naïve rats were present in a population. In fact, both *S. ratti* and *S. venezuelensis* are common parasite of wild rats, therefore suggesting that sterile anti-*Strongyloides* immunity does not commonly develop in the wild. Wild animals do not appear to be generally immuno-impaired compared with their laboratory cousins (Abolins *et al.* 2011; Viney *et al*. 2015), suggesting that the apparently better survival of *S. ratti* in wild rats is either because most infections are very low intensities (thus not inducing a Th2 response) and/or that the anti-*S. ratti* immune responses in wild rats is different compared with that of laboratory rats. Of note, long-term experiments with *S. ratti* in which infections were established with single iL3s, showed that these infections lasted in excess of 6 months (Graham, 1938*a*, *b*, 1940*a*, *b*).

## GENETIC VARIATION FOR INFECTION TRAITS

Comparison of different *S. ratti* isofemale lines has shown that they differ in their survival within the host and in their *per capita* fecundity, particularly as the host immune response develops (Paterson and Viney, 2003). This suggests that different isofemale lines are differently affected by the host immune response with respect to the life-history traits of survival and reproduction. These data also suggested that there may be a trade-off between the lines’ survival and reproduction (Paterson and Viney, 2003), as is commonly seen among other taxa.

## BIOLOGY OF THE FREE-LIVING GENERATION

The free-living generation of *S. ratti* consists of a single adult generation (Yamada *et al*. 1991; Viney and Lok, 2015) that contains a developmental choice. Specifically, eggs passed from an infected host are genetically male or female, and female larvae can choose to develop directly into iL3s (ready to infect a new host) or they can develop into adult free-living females (the so-called indirect development) (Viney, 1996; Viney and Lok, 2015).

The free-living adult generation of *S. venezuelensis* is also known and was described at the species’ second description (Little, 1966). However, in many *S. venezuelensis* isolates no free-living males are observed (Hino *et al*. 2014; A. Muro, personal communication), and therefore any free-living females present will not be fertilized, and so only directly developing iL3s will be produced. This phenomenon may be an artefact of long-term laboratory maintenance of *S. venezuelensis*, and/or variation among different wild sources. In support of the latter hypothesis even fresh, wild *S. venezuelensis* isolates have been found to not have any free-living males, and free-living females only rarely (Hasegawa *et al*. 1988). Long-term laboratory maintenance of *S. ratti* has not resulted in a similar decline in the free-living adult generation. Isofemale lines of *S. ratti* that developed by mixed direct and indirect development have been successfully artificially selected into separate lines where direct or indirect development dominated (Viney, 1996). There was a rapid response to this selection, which is consistent with the diversity of patterns of development seen among wild-derived isofemale lines.

In *S. ratti* the direct *vs* indirect female-only choice is affected by the worms’ environment. A number of factors have been identified that affect this choice in *S. ratti* as well as in other *Strongyloides* spp. (Graham, 1939; Viney, 2002). In *S. ratti*, an interaction of the immune response of the host from which the developing eggs came and the temperature external to the host are key to control of the development of the free-living generation (Harvey *et al.* 2000). The host immune response promotes indirect development of free-living females, as does a higher external temperature. The interaction of these factors is seen as female eggs/developing larvae being more sensitive to temperature as the immune response of their host increases (Harvey *et al.* 2000). The sex ratio of the progeny of the parasitic females is also affected by the host immune response, with a more male-biased ratio occurring in the presence of a host immune response. Together the host immune response therefore promotes the development of the free-living adult generation. In laboratory infections the host immune response increases as the infection progresses, so that indirect development *via* free-living adults is increasingly favoured at the expense of direct development during an infection (Viney *et al*. 1992; Viney, 1996).

This is a finding with important consequences. Firstly, it means that developing female larvae developmentally respond to the host immune response. There are other examples of parasitic nematodes specifically responding to the host immune response (Viney, 2013), but the response of *S. ratti*'s larvae to this host cue occurs external to the host. Secondly, that developing larvae integrate different cues (the host immune response and temperature) in making their developmental choice (Crook and Viney, 2005). Thirdly, that these cues are separated in space and time, implying that these worms have some way of remembering information (Viney, 2013). This could be by a direct neuronal mechanism (perhaps analogous to the ability of *Caenorhabditis elegans* adults to remember the temperature at which they were fed as larvae; Ardiel and Rankin, 2010), by the host immune response affecting the local faecal environment of the larvae, or the larvae's parasitic female mothers epigenetically setting the developmental course of their progeny. These three possibilities for larval memory are not mutually exclusive.

The developmental pattern of the free-living generation of *S. ratti* varies among genotypes (Viney *et al*. 1992). Specifically, comparison of different *S. ratti* isofemale lines shows that they differ in the degree of direct *vs* indirect development. Isofemale lines derived from isolates obtained in the UK develop almost exclusively by direct development. Isofemale lines from Japan and the USA have mixed direct and indirect development (Viney *et al*. 1992). Because the development of the lines changes as the host immune response develops, then these developmental differences of the lines also show that lines vary in their developmental sensitivity to the host immune response. The adaptive value of different developmental strategies is not clear, but the observed diversity is presumably some reflection of adaptation to the local ecology and epidemiology of where these *S. ratti* genotypes originated (Fenton *et al*. 2004). Note, the among-line variation in free-living development must also be considered with the context of the among-line variation in the immune-dependent survival and fecundity of the parasitic female generation (see above; Paterson and Viney, 2003).

## THE *STRONGYLOIDES* FREE-LIVING GENERATION AND THE FREE-LIVING NEMATODE DAUER LARVA ANALOGY

The infective larvae of parasitic nematodes have long been considered to be analogous to the dauer larvae of free-living nematodes (most thoroughly studied in *C. elegans*), and that such dauer larvae were a key step in the evolution of nematode parasitism (Hotez *et al*. 1993). Dauer larvae are developmentally arrested, non-feeding, third stage larvae. In free-living nematodes such as *C. elegans*, young larval stages have a choice between developing into dauer larvae or continuing to grow into reproductive adults (Riddle and Albert, 1997). The conventional understanding with *C. elegans* is that conditions of environmental stress can favour the development of dauer larvae; more recent results suggest that the drivers of dauer larva formation are more complex and diverse than a simple environmental stress response, and may be driven by a range of complex ecological interactions (Viney and Diaz, 2012; Diaz *et al.* 2014).

The free-living phase of the *Strongyloides* life-cycle particularly invites comparison with the life-cycle and developmental choice of free-living nematodes, and molecular confirmation of this analogy has been sought. The molecular control of the initiation of *C. elegans* dauer larva development is well known. Key to this developmental choice is the gene *daf-7*, encoding a *C. elegans* TGF-β ligand such that DAF-7 activity supresses *C. elegans* dauer larva formation (Ren *et al*. 1996). In *C. elegans*, the expression of *daf-7* is high in first stage larvae (L1s), but it is not expressed in dauer-destined L2s, dauer larvae, or adults. If the developmental choice of *S. ratti* and *C. elegans* were molecularly conserved, the expression of the *daf-7* homologues of *C. elegans* and *S. ratti* would be predicted to be conserved too. However, when investigated this pattern was not found; instead, in *S. ratti daf-7* has maximum expression in iL3s, and this expression is immediately reduced upon their exposure to hosts (Crook *et al*. 2005). This pattern was also seen in *Parastrongyloides trichosuri*, and analogous findings were made in hookworms and filarial nematodes (Gomez-Escobar *et al*. 2000; Brand *et al*. 2005; Freitas and Arasu, 2005). Together this suggests that in parasitic nematodes *daf-7* has evolved to have a role in the control of the infective larval stage of parasitic nematodes (Gomez-Escobar *et al*. 2000; Brand *et al*. 2005; Freitas and Arasu, 2005; Viney *et al*. 2005). This makes some considerable sense because in free-living nematodes the critical life-cycle choice is between dauer or non-dauer development, whereas in parasitic nematodes the key choice is for infective larvae to decide whether they have met the correct host conditions (so that they can resume their development and infect the host), or whether they have not (and so they remain developmentally arrested, continuing to seek the correct host). It is perhaps for this reason that in parasitic nematodes *daf-7* has been used in this key developmental choice of infective larvae, rather than in the formation of infective larvae *per se.*

## COMPARATIVE LIFE-HISTORY BIOLOGY OF THE TWO ADULT GENERATIONS

The life-cycle of *Strongyloides* has two adult generations: one parasitic, female-only and parthenogenetic and one free-living, dioecious and sexually reproducing. The comparative life-history of these two adult phases can give us an insight into likely selection pressures operating on these different life-cycle phases. Comparison of the ageing biology of these phases is particularly instructive. The evolutionary theory of ageing explains that a species with high extrinsic mortality rate will evolve a shorter lifespan compared with one with a low extrinsic mortality rate (Gardner *et al.* 2006). This prediction has been well supported by many among-taxa comparative studies.

In *S. ratti*, the lifespan of the two adult female forms is *c.* 80-fold different (Gardner *et al.* 2006). Parasitic females in an immuno-incompetent host live for over a year before they die, apparently due to senescence (Gardner *et al.* 2006). In contrast, the free-living females live a maximum of 5 days when maintained in apparently optimized conditions (Gardner *et al*. 2004). [And the life-time fecundity of parasitic female (*c.* 16 000) is 400-fold greater than that of the free-living females (*c.* 40) (Gardner *et al.* 2006; Thompson *et al.* 2009).] These two vastly different lifespans are controlled from one genome, showing that this lifespan plasticity is epigenetically controlled.

The vastly different lifespans of these two forms can therefore tell us about the relative extrinsic mortality rates of the parasitic and free-living female morphs. Specifically, that the free-living females likely have a comparatively very high extrinsic mortality rate compared with the parasitic females (Gardner *et al.* 2006). This may, initially, be counter intuitive, because we tend to think of a within-host environment (containing an active host immune response etc.) as hostile and unpleasant. In fact, these results suggest that it is a comparatively more benign environment compared with a free-living environment, perhaps because a within-host environment is much more predictable and resource rich, to which a species or life-cycle morph can adapt and specialize.

## *STRONGYLOIDES RATTI* AND *S. VENEZUELENSIS* AS MODELS OF STRONGYLOIDIASIS IN HUMANS

Parasitic nematodes, including *Strongyloides*, are important pathogens of humans and other animals. For this reason, there is considerable interest in developing amenable animal models of infection. In this context *S. ratti* and *S. venezuelensis* infections of mice have been used, particularly for immunological studies. An apparently unique feature of human *S. stercoralis* infection is the occurrence of internal autoinfection (Lok *et al*. 2016). This phenomenon does not naturally occur with *S. ratti* or *S. venezuelensis*, and neither can it be artificially induced. Therefore, neither of these species are models that recapitulate this aspect of human strongyloidiasis, meaning that neither of these parasite of rodents are particularly good models for human strongyloidiasis.

## OUTSTANDING QUESTIONS

The existence of two species of *Strongyloides* in rats, but the apparent absence of a species naturally infecting mice is an intriguing feature of the natural history of the *Strongyloides* genus. That each of these two species is from different sub-clades of *Strongyloides* argues that *S. ratti* and *S. venezuelensis* represent two independent evolutionary transitions to parasitism of rats. This accident of natural history presents an ideal opportunity to study the biology of nematode host-specificity, since features (physiological, genetic, etc.) of *S. ratti* and *S. venezuelensis* that are concordant, but which are also discordant compared with other respective close *Strongyloides* spp. relatives parasitizing other host species, can identify what it takes for *Strongyloides* spp. to specifically parasitize rats. There has been one, ultimately unsuccessful, attempt to artificially select *S. ratti* to change its host specificity (Gemmill *et al.* 2000).

Further, there has been considerable recent interest in parasite co-infections (Viney and Graham, 2013) and the existence of two con-generic species infecting rats (along with the ability of other infections to be introduced), presents a superb opportunity to unpick many key aspects of co-infection biology. Also, because *S. ratti* appears to be ubiquitous in the wild, but *S. venezuelensis* more restricted, then different wild sources of *S. ratti* will have evolved in different co-infection contexts, particularly with respect to *S. venezuelensis* co-infection. Comparative study of *S. ratti* from these different settings could therefore be used to understand how co-infection drives the evolution of parasite traits. With respect to *Strongyloides* and the developmental choice of its free-living cycle, it would also be interesting to understand how the co-infection context of these two species affects their developmental choices.

In conclusion, *S. ratti* and *S. venezuelensis* are common parasites of rats and tractable laboratory systems. There is now a good understanding of their within-host biology showing that while these species are broadly similar in their biology there are clear, notable differences. The biology of the free-living generation of *S. ratti* is now well understood, and it is clear that the free-living generation is of less importance for *S. venezuelensis.* The recent genomic, transcriptomic and proteomic analysis of these and other *Strongyloides* spp. (Hunt *et al.* 2016) now provides a firm platform for investigating the molecular basis of key aspects of *Strongyloides* biology.
